# On Placement, Location and Orientation of Wrist-Worn Tri-Axial Accelerometers during Free-Living Measurements

**DOI:** 10.3390/s19092095

**Published:** 2019-05-06

**Authors:** Marcin Straczkiewicz, Nancy W. Glynn, Jaroslaw Harezlak

**Affiliations:** 1 Department of Epidemiology and Biostatistics, School of Public Health-Bloomington, Indiana University, Bloomington, IN 47405, USA; harezlak@iu.edu; 2 Center for Aging and Population Health, Department of Epidemiology, Graduate School of Public Health, University of Pittsburgh, Pittsburgh, PA 15213, USA; glynnn@edc.pitt.edu

**Keywords:** wearable computing, sensor position, physical activity, ActiGraph GT3X+

## Abstract

Wearable accelerometers have recently become a standalone tool for the objective assessment of physical activity (PA). In free-living studies, accelerometers are placed by protocol on a pre-defined body location (e.g., non-dominant wrist). However, the protocol is not always followed, e.g., the sensor can be moved between wrists or reattached in a different orientation. Such protocol violations often result in PA miscalculation. We propose an approach, PLOE (“Placement, Location and Orientation Evaluation method”), to determine the sensor position using statistical features from the raw accelerometer measurements. We compare the estimated position with the study protocol and identify discrepancies. We apply PLOE to the measurements collected from 45 older adults who wore ActiGraph GT3X+ accelerometers on the left and right wrist for seven days. We found that 15.6% of participants who wore accelerometers violated the protocol for one or more days. The sensors were worn on the wrong hand during 6.9% of the days of simultaneous wearing of devices. During the periods of discrepancies, the daily PA was miscalculated by more than 20%. Our findings show that correct placement of the device has a significant effect on the PA estimates. These results demonstrate a need for the evaluation of sensor position.

## 1. Introduction

Accelerometers have become an increasingly popular tool for the objective assessment of physical activity (PA). Currently, we use accelerometers both for laboratory experiments and in a free-living environment. The latter is recognized as particularly beneficial to studies considering the evaluation of human behavior [1]. In early free-living studies, accelerometers were typically located close to the center of body mass, e.g., on the waist, hip or lower back [2]. However, recent large cohort studies, such as UK Biobank and U.S. National Health and Nutrition Examination Survey (NHANES) [3,4], used sensors placed on a wrist following suggestions that this body placement improves wear compliance, particularly among younger participants and during prolonged measurements [4,5].

In unsupervised experiments, activity monitors are most frequently placed in a pre-defined position that remains the same during the measurement [6]. Nevertheless, this requirement may not always be satisfied, e.g., the sensor can be moved from the left to the right wrist or reattached so that the device has the opposite orientation [7,8]. If left unaccounted for, these experimental protocol violations can lead to considerable PA miscalculation during the period of discrepancy [7,9].

Several methods have been proposed to address the issue of the device’s altered orientation, e.g., by rotating the tri-axial measurement to the common domain or by providing an orientation-invariant classification technique [9,10]. However, to the best of our knowledge, there are no published methods investigating changes of sensor location during the free-living measurements.

A major goal of our work is to establish the participants’ compliance with the study protocol. To achieve it, we developed a method assessing location, placement and orientation of the wrist-worn tri-axial accelerometers during the free-living measurements. Our approach, called “PLOE”, short for “Placement, Location and Orientation Evaluation method”, considers sensor location (left or right wrist), placement (top or bottom of a wrist), and orientation (wristwatch-like or upside-down position). PLOE is based on comparing the spatial orientation of the device during standing and sitting postures with the expected orientation based on the position provided by the protocol. We apply our method to the measurements collected from the ActiGraph GT3X+ accelerometers worn on both wrists for 7 days of free-living activities. We use an activPAL device placed on a thigh to provide the ground truth about body posture. The experiment was conducted at the University of Pittsburgh on a cohort of older adults (N = 45). We report the number of days when the devices were worn against the protocol. In addition, we quantify the impact of sensors’ misplacement on PA estimates.

## 2. Materials and Methods

### 2.1. Study Sample and Devices

Forty-five community-dwelling older adults (23 men and 22 women) were recruited from the Pittsburgh, Pennsylvania area to participate in the Aging Research Evaluating Accelerometry (AREA) project, part of the Development Epidemiologic Cohort Study (DECOS). The AREA project was aimed at examining the impact of accelerometers’ wear location on the assessment of PA among older adults. The study was approved by the Institutional Review Board of the University of Pittsburgh; all participants provided written informed consent. All participants were in good overall physical health and reported no current history of adverse medical conditions that could affect free-living activities. The detailed description of the inclusion and exclusion criteria for DECOS can be found elsewhere [11,12].

The study involved a 7-day continuous collection of free-living data. Measurements were obtained using 3 portable accelerometers: 2 ActiGraph GT3X+ devices and 1 activPAL device. The ActiGraph sensors (ActiGraph Inc., Pensacola, FL, USA) were placed on the left and the right wrists. In the described cohort, a total of 20 ActiGraph GT3X+ sensors were used. They collected data in 3 orthogonal axes with a sampling frequency equal to 80 Hz and a dynamic range of ±6 g. The third sensor, activPAL (PAL Technologies Ltd. Glasgow, UK), was affixed to the left thigh using a hypoallergenic medical dressing following the recommended position of about one-third of the way down the thigh along the midline. It provided indications of body posture (sitting/lying vs. standing) aggregated into 15 s epochs with 1 s precision. ActivPAL was recognized as a valid tool for estimation of body posture in free-living measurements [13,14], and hence in our investigation it provided ground truth on periods of lying/sitting and standing postures.

### 2.2. Data Cleaning

The collected data were examined for non-wear time using the procedure outlined by van Hees and colleagues [15]. We investigated the dataset of raw accelerometry measurements using a sliding 30 min window and defined as non-wear any measurement fragment with standard deviation of acceleration less than 13 mg and value range less than 50 mg for at least 2 out of 3 axes. We repeated this step for all monitors. To align measurements across various body locations, any non-wear period of an individual device was simultaneously treated as non-wear for the remaining ones. Next, for each measurement day, we calculated total wear time. A valid wear day consisted of at least 10 h of valid data [16]. As a result, the preprocessed dataset included 32 participants with 7 valid days, 9 participants with 6 valid days, 1 participant with 5 valid days and 3 participants with 3 valid days. Altogether, 292 measurement days were used in further analysis.

In the next step, we used the aggregated recordings of activPAL and limited the dataset to periods when activPAL indicated either 15 s of standing or 15 s of sitting/lying body posture. The intermediate periods, containing, e.g., 4 s of upright and 11 s of sitting/lying posture, were excluded from the analysis. This step was mandatory since the activPAL-associate software does not provide information about time and number of body transitions within the epoch. Finally, to increase the probability of sitting/lying periods to consist entirely of a sitting posture, we limited the dataset to the daytime periods defined as between 7 am and 11 pm [17].

### 2.3. PLOE—Placement, Location and Orientation Evaluation Method

#### 2.3.1. Spatial Inclination of Tri-Axial Accelerometers

**Figure 1:**
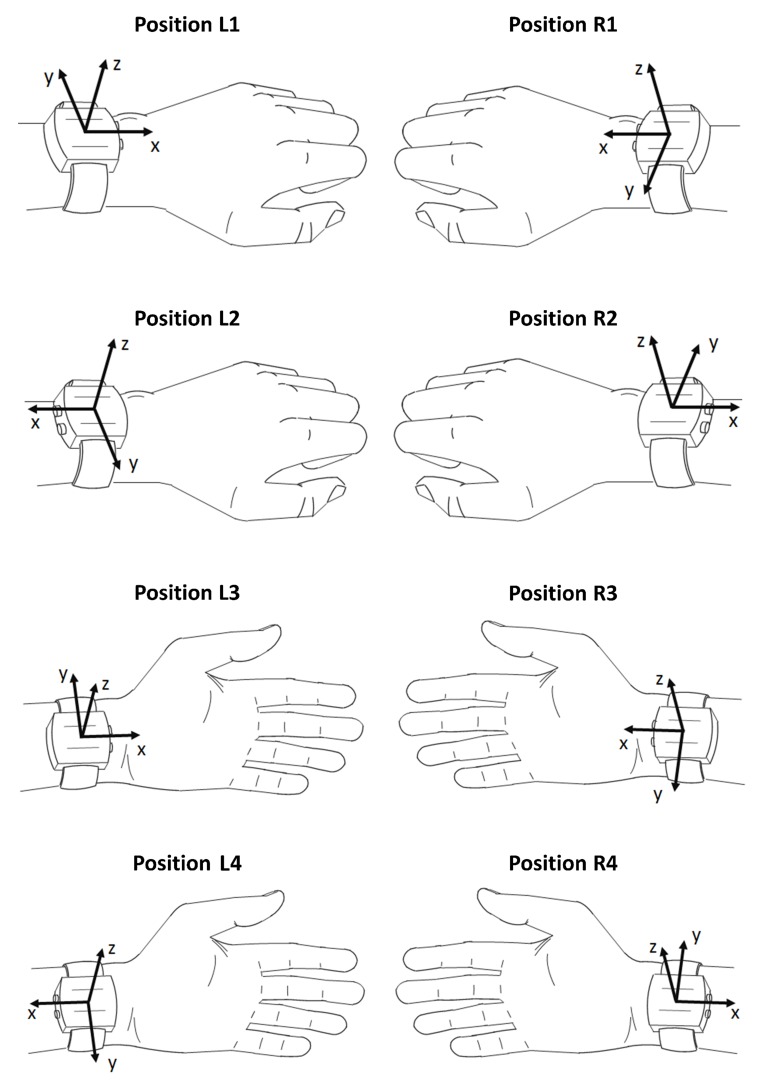
Eight positions of the wrist-worn wearable accelerometer displayed with the coordinate system on the device.

Standard accelerometers provide measurements in 3 orthogonal axes: anteroposterior (x-axis), mediolateral (y-axis) and vertical (z-axis) [18]. When the device is worn on a wrist, the x-axis corresponds to the direction parallel to the forearm; y-axis, perpendicular to the forearm and parallel to the skin surface, and z-axis, perpendicular to the skin surface (Figure 1).

As an output, accelerometers provide a combination of dynamic and static accelerations in the given axis of measurement [19]. Whilst the dynamic acceleration reflects temporal body movements, the static acceleration informs about sensor inclination relative to the gravitational vector and, hence, about the spatial orientation of the device. Therefore, if the accelerometer is placed on a flat horizontal surface, its measurement axes indicate +1, −1 or 0 g depending on the same, the opposite or the perpendicular direction to the gravitational vector, respectively. To retrieve the spatial inclination from the raw accelerometry data, it is common to use a low-pass filter with a cut-off frequency between 0.1 and 0.5 Hz [19] or a median filter of high order (i.e., the moving median with a long window) [20].

#### 2.3.2. Sensor Position

The position of a wrist-worn accelerometer is characterized by its location, placement and orientation. The location describes whether a sensor is worn on the left or on the right hand. On each hand, the accelerometer can be affixed in 2 distinctive placements: on the top of a hand, like a wristwatch, and on the bottom of a hand. Furthermore, in each placement the accelerometer can be oriented in 2 ways: with the x-axis directed toward the palm or toward the elbow. Since by design, most research-grade activity monitors (e.g., ActiGraph GT3X+ or GENEActiv, Activinsights Ltd, Kimbolton, UK) are not equipped with a display screen, each pair of orientations can be used interchangeably without influence on the wear comfort or on the functionality of the device. While the sensor placement and orientation are subject-specific, the location is imposed by study objectives, e.g., investigation of non-dominant wrist movements.

Cumulatively, there are 4 combinations of sensor placements and orientations per wrist (Figure 1). The combinations for the left wrist are presented in the left column, while the combinations for the right wrist are displayed in the right column. To simplify the description in the following sections, the devices worn on the top of a left or right hand with the x-axis directed toward the palm are referred to as being carried in position L1 or R1, respectively (top plots). In positions L2 or R2, the device is worn on the top of a left or right hand, respectively, with the x-axis directed toward the elbow (middle top plots). In positions L3 or R3, the device is worn on the bottom of a left or right hand, respectively, with the x-axis directed toward the palm (middle bottom plots). Finally, in positions L4 or R4, the device is worn on the bottom of a left or right hand, respectively, with the x-axis directed toward the elbow (bottom plots).

#### 2.3.3. Key Concepts and the Algorithm

To determine the sensor’s location, placement, and orientation, we leverage observation on the dominant hand position in 2 body postures, namely standing and sitting. When a person is standing, his hands are predominantly directed downwards or are in the transition between horizontal and vertical planes. In turn, while sitting, a person’s hands are predominantly oriented horizontally and arranged so that his thumbs are directed somewhere between upwards and toward the sagittal plane of the body. These observations are reflected in the static acceleration of tri-axial accelerometers, principally in the indications of the x-axis when a person is standing, and in the indications of y- and z-axes when a person is sitting.

For instance, when the device is worn in position L1 by a standing person, the acceleration measured along the x-axis is equal to +1 and 0 g for the vertical and horizontal position of a hand in its resting position, respectively. If the device is rotated by 180° around the z-axis, i.e., it is carried in position L2, the corresponding x-axis accelerations are equal to −1 and 0 g. The same reasoning can be applied for accelerations from y- and z-axes during sitting. In position L1, the direction of the y-axis is opposite to the thumb. Therefore, when the left-hand thumb is directed upwards, the y-axis is directed downwards and hence it indicates +1 g. In this circumstance, the z-axis is parallel to the ground surface, thus its static acceleration is equal to 0 g. When the left-hand thumb is directed toward the sagittal plane of the body, the z-axis indicates −1 g (directed upwards), while the y-axis indicates 0 g (directed horizontally). In position L2, the y-axis is aligned with the thumb, thus when the left-hand thumb is directed upwards, so is the y-axis indicating −1 g. In such a scenario, the z-axis is perpendicular to the ground, thus it indicates 0 g. When the left-hand thumb rotates to the sagittal plane, the z-axis indicates −1 g, while the y-axis indicates 0 g. To account for frequent hand movements, which are the norm in real-life conditions, in PLOE we examine axes regarding the predominant hand orientation. Therefore, in position L1 the static accelerations of the x-axis are predominantly positive (>0 g), so are the indications of the y-axis, while the z-axis is predominantly negative (<0 g). Consequently, in position L2 the indications of all axes are negative.

In the proposed method, PLOE, we determine the sensor position using the indications retrieved from the raw accelerometry measurements collected during the 7-day free-living activity. We calculate median acceleration from the x-axis when a person is standing, and median accelerations from the y- and z-axes when a person is sitting. If the monitor is worn in position L1, we expect signs of median x- and y-axis accelerations to be positive and the sign of z-axis acceleration to be negative (Table 1). In turn, when the device is worn in position L2, we expect all the signs to be negative. Obtained combinations of signs determine the actual sensor position. Our method benefits from the fact that there is a unique combination of signs of medians for each location, placement and orientation of a wrist-worn accelerometer. We summarize these combinations in Table 1. Finally, we compare the estimated position with the study protocol and identify discrepancies. The full PLOE algorithm including data preprocessing is as follows:

1. Exclude non-wear periods.
2. Find periods of standing and sitting.
3. For each day, calculate median acceleration in the x-axis for periods of standing and median accelerations in y- and z-axes for periods of sitting.
4. In Table 1, find a combination of signs from Step 3, which is the estimated sensor position.
5. The compliance is true if the actual sensor position from Step 4 is the same as in the study protocol.

### 2.4. Estimation of Physical Activity (PA)

Sensor misplacement (e.g., from non-dominant to dominant wrist) can affect the PA estimates. To investigate its impact, we calculate signal mean absolute deviation (*MAD*), a good proxy of PA intensity levels [21]. *MAD* is defined as the mean absolute deviation of the acceleration signal:

(1) $$MAD=\frac{1}{T}\sum_{t=1}^{T}\left|VM_{t}-\;\frac{1}{T}\sum_{t=1}^{T}VM_{t}\right|,$$

where *T* denotes the total number of samples for the time period where *MAD* is computed and $VM_{t}$ denotes the instantaneous vector magnitude of the tri-axial acceleration signal. We calculate *MAD* in non-overlapping one-minute intervals and summarize it for each measurement day and for each wrist. The impact of sensor misplacement is assessed as a percentage difference between *MAD* calculated from the intended and estimated sensor location. We report mean, standard deviation (SD), minimum and maximum percentage difference and 95% confidence intervals of the population level difference. It is important to note that common PA estimates are invariant to sensor orientation and placement. Hence, any sensor rotation, e.g., from the top to the bottom of a selected hand, does not impact on such metrics. All calculations were performed in the MATLAB environment.

## 3. Results

We analyzed data from 45 participants who had complete accelerometry data acquired during the free-living activities over a 7-day time period. Participants were between 70 and 90 years of age (mean = 78.0, SD = 5.5), body mass index ranged between 20.5 and 37.9 kg/m^2^ (mean = 25.8, SD = 3.7), and height between 153.2 and 184.2 cm (mean = 166.3, SD = 7.7). Forty-two participants were right-handed and the remaining were left-handed. The average daily *MAD* for non-dominant and dominant hands were equal to 32.6 g (SD = 11.6) and 36.8 g (SD = 13.6), respectively. The overall correlation between daily *MAD* of non-dominant and dominant wrists was strong (R^2^ = 0.94, *p*-value < 0.001).

### 3.1. Evaluation of Sensor Position

The evaluation of sensor position revealed that 7 out of 45 (15.6%) participants wore the sensor on the opposite hand to the one defined in the study protocol. The hand change was observed during 20 (6.9%) days of simultaneous wearing of devices. Moreover, one additional participant changed sensor orientation for one day.

**Figure 2:**
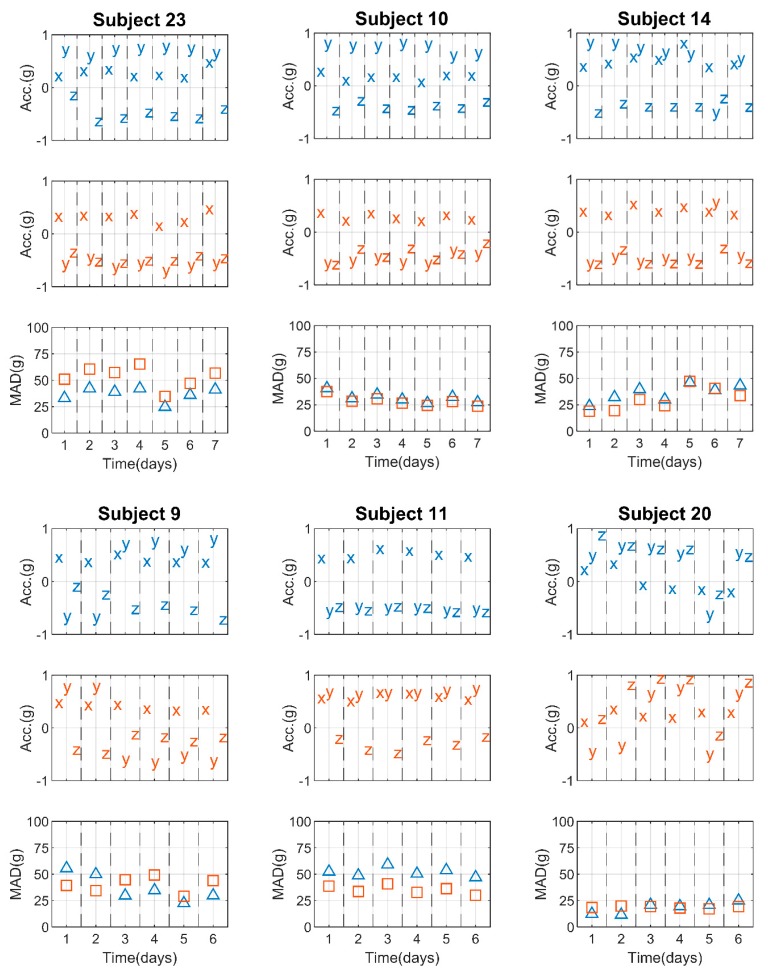
Plots of daily median accelerations of left (blue) and right wrists (red) corresponding to daily summed minute-to-minute *MAD* (blue triangles and red squares, respectively). The indications are presented for 6 selected participants, where measurements for Subject 23 and 10 were acquired correctly, and measurements for the remaining participants exhibited various discrepancies, primarily the switch of sensor placement from left to right wrist and vice versa for one day (Subject 14), for 2 days (Subject 9 and Subject 20) or for 6 days (Subject 11). Additionally, for Subject 20 the devices were worn in various orientations.

To provide a better understanding of the method, in Figure 2 we present scatterplots of tri-axial medians and *MAD* corresponding to particular days of measurement of selected subjects. Displayed in blue and red are the indications from the left and right wrist, respectively. All the selected participants, except Subject 10, were right-handed.

Among the selected subjects, there were 2 participants, namely Subjects 23 and 10, who followed the study protocol throughout the entire measurement, i.e., sensors intended for left and right wrists were worn accordingly, specifically in positions L1 and R2, respectively. This was estimated by predominantly positive signs of x-axis medians, positive and negative signs of y-axis medians for the left and right wrist, respectively, and negative z-axis medians, which indicates that the sensors were placed on top of hands for 7 days.

Some aberration of sensor indications can be seen in the measurements from Subject 14. In this case, during the 6th day of the experiment, the sign of the y-axis median changed while the signs of the x and z-axis medians remained the same. This suggests a one-day misplacement of accelerometers from left wrist (position L1) to right wrist (position R2) and vice versa. A similar observation can be made for Subject 9, where the potential switch of hands concerned the 2 initial days of the data acquisition.

For Subject 11, constantly negative and positive y-axis medians for the left and right wrists, respectively, combined with the positive x-axis medians and negative z-axis medians, suggest that the participant wore the sensors on opposite hands for the entire duration of the experiment.

Another example of sensor position deviation was observed in Subject 20. During 5 out of 6 days of measurement, the sensors were likely placed on the bottom side of the hand, which was revealed by positive z-axis medians during sitting. The investigations of x- and y-axes indicated that the device intended for the left wrist was worn in position R3, i.e., on the opposite wrist, while the accelerometer that was intended for the right wrist was worn in position L3. A change of hand was not the case during the following days, since the signs of the median provided by each sensor followed the protocol, however their position was altered. According to our algorithm output, the sensors were carried in the following manner: in positions L4 and R3 during days 3, 4 and 6, and in positions L2 and R1 during day 5, for the left and right wrist, respectively (Table 2).

### 3.2. Impact of Sensor Location Change on the PA Estimates

Another aspect of the investigation concerned the comparison between *MAD* calculated from the non-dominant and dominant wrist regarding their intended and estimated position. For accelerometers originally intended for the non-dominant wrist and subsequently identified as worn on the dominant wrist, on average, the daily level of PA measured by *MAD* was overestimated by 22.6% (SD = 17.8, min = 4.2, max = 71.4, 95%CI = 14.7 − 30.4). In turn, when the sensor was supposed to be worn on the dominant wrist and was actually worn on the opposite hand, on average, it resulted in underestimation of daily *MAD* by 25.9% (SD = 19.8, min = 4.1, max = 57.1, 95%CI = 17.2 − 34.6).

The shifted indications of *MAD* (Figure 2) revealed the sensor misplacement. In addition, when a sensor was worn according to the protocol, the daily *MAD* was usually higher for one of the wrists. In turn, during the detected days of misplacement this relation was reversed, as for Subject 9 and Subject 20.

## 4. Discussion

We evaluated our newly proposed method, PLOE, to assess the placement, location and orientation of wrist-worn tri-axial accelerometers during free-living activity measurements. Since accelerometers became a standalone tool to assess PA, it is crucial that the collected measurements and their protocol description are as cohesive as possible. Special attention needs to be given to free-living data collection, where researchers have limited control over the measurement process. This requires careful planning of the data acquisition and pre-processing procedures. As reported in [4], one way to increase the compliance is to place the sensor on the wrist rather than on the waist. Another aspect has to do with the way the sensor is affixed. It is suggested to be comfortably tight and protected from reattachment [22]. However, in real-life conditions a sensor might loosen up or be taken off (e.g., for bath or night-time) and reattached in a different position. In such cases, the pre-processed recording might be considered as valid in terms of wear-time but it is incorrect in terms of the sensor location described in the study protocol. Considering the fact that PA classification systems are typically designed for a given sensor position, such protocol deviations can lead to a significant PA estimation error. In our investigation, we estimated that about 16% of study participants did not comply with the protocol and changed the device location for at least one measurement day. Although this suggests that a large portion of the study sample was collected incorrectly, the total number of days with discrepancy was estimated to be about 7%.

As presented, changes in sensor location had a considerable impact on PA estimates. This was revealed by a miscalculation of daily *MAD* by more than 20% on average. These results demonstrate the need for the evaluation of sensor position. They also provide additional insight into differences between cumulative acceleration of dominant and non-dominant wrists, especially in the context of previous studies. For instance, in [23] the authors found no significant difference between wrists’ accelerations. Similar results were presented in [24], however both studies investigated cohorts of young adults (mean age was approximately 32 and 22 years, respectively). The preceding research on an older sample (mean age was approximately 49 years [25]) and a more exhaustive study on hand-dependent accelerations of AREA participants [26] suggest that the difference between hand use might be related to age, and therefore this requires further examination. Regardless of these findings, the investigation of sensor position should also be undertaken prior to the application of machine learning techniques and activity recognition algorithms that utilize measurements from specific axes.

The presented study has a number of limitations. First, a true gold standard of the actual sensor position is not available and we rely on the estimated body position based on activPAL measurements. This limitation, however, is not unique to our study as studies conducted in the free-living environment very frequently rely on “silver standards”. Second, the use of 2 lookalike wrist-worn monitors might have confused participants during the sensor reattachment and thus influenced the number and frequency of sensor misplacements. Third, in our approach the ground truth on body posture was provided by activPAL while in many studies this device is not utilized. One of the feasible solutions to overcome this obstacle is to employ an activity recognition technique validated for wrist-worn accelerometers that uses signal vector magnitude rather than individual axes [27,28].

The proposed method is dedicated to sensors located on the left and right wrist. In the presented investigation, we monitored their changed position in one-day intervals. The selection of this time window was driven by a trade-off between the precision of misplacement detection and the daily predominant hand positioning during standing and sitting. We observed that, while standing, hands are typically pointing downwards. Meanwhile, when sitting, hands are typically oriented horizontally so that the thumbs are between the upright and toward the sagittal plane of the body. Nevertheless, during selected activities, such as talking on a phone while standing (hand pointed upwards) or holding a phone while sitting (thumbs against the sagittal plane), the described observations are not present. Moreover, in real life measurements the frequency and duration of such events is unknown. Therefore, to reduce the effect of this limitation on the accuracy of PLOE, we expanded the time window to one day and hence limited its influence on the investigated features.

In conclusion, whilst our findings suggest that switching sensor location is not very frequent, the significant implications of sensor misplacement should lead to further research in this field. The identified research directions should primarily involve the inclusion of a true gold standard of the actual sensor position and more sensor locations. The former goal can be accomplished by the post-experiment survey inspecting changes of sensor position. Alternatively, a study could include body-worn cameras that capture video frames during everyday activities [29]. The latter goal is motivated by the common and widespread PA estimation methods that involve the waist and hip (e.g., [30,31]). We hypothesize that the estimation of sensor position on other body locations can be accomplished using pattern recognition techniques that measure similarities between features of selected activities, e.g., walking.

## Figures and Tables

**Table 1:** Expected signs of median accelerations provided by tri-axial wrist-worn accelerometers during sitting and upright body posture.

Sensor Position	L1	L2	L3	L4	R1	R2	R3	R4
**x-axis (upright)**	+	-	+	-	+	-	+	-
**y-axis (sitting)**	+	-	-	+	-	+	+	-
**z-axis (sitting)**	-	-	+	+	-	-	+	+

**Table 2:** Intended and actual position of accelerometers during the measurement of Subject 20.

Day of Measurement	1	2	3	4	5	6	1	2	3	4	5	6
**Predefined position**	L1						R1					
**x-axis (upright)**	+	+	-	-	-	-	+	+	+	+	+	+
**y-axis (sitting)**	+	+	+	+	-	+	-	-	+	+	-	+
**z-axis (sitting)**	+	+	+	+	-	+	+	+	+	+	-	+
**Actual position**	R3	R3	L4	L4	L2	L4	L3	L3	R3	R3	R1	R3
